# Fractionation of Raw and Parboiled Rice Husks with Deep Eutectic Solvents and Characterization of the Extracted Lignins towards a Circular Economy Perspective

**DOI:** 10.3390/molecules27248879

**Published:** 2022-12-14

**Authors:** Chiara Allegretti, Emanuela Bellinetto, Paola D’Arrigo, Monica Ferro, Gianmarco Griffini, Letizia Anna Maria Rossato, Eleonora Ruffini, Luca Schiavi, Stefano Serra, Alberto Strini, Stefano Turri

**Affiliations:** 1 Department of Chemistry, Materials and Chemical Engineering “Giulio Natta”, Politecnico di Milano, p.zza L. da Vinci 32, 20133 Milano, Italy; 2 Istituto di Scienze e Tecnologie Chimiche “Giulio Natta”, Consiglio Nazionale delle Ricerche (SCITEC-CNR), Via Luigi Mancinelli 7, 20131 Milano, Italy; 3 Istituto per le Tecnologie della Costruzione, Consiglio Nazionale delle Ricerche (ITC-CNR), Via Lombardia 49, 20098 San Giuliano Milanese, Italy

**Keywords:** deep eutectic solvent (DES), lignocellulosic biomass fractionation, raw rice husk, parboiled rice husk, parboiling process, lignin, cement plasticizers, sustainability, circular economy

## Abstract

In the present work, rice husks (RHs), which, worldwide, represent one of the most abundant agricultural wastes in terms of their quantity, have been treated and fractionated in order to allow for their complete valorization. RHs coming from the raw and parboiled rice production have been submitted at first to a hydrothermal pretreatment followed by a deep eutectic solvent fractionation, allowing for the separation of the different components by means of an environmentally friendly process. The lignins obtained from raw and parboiled RHs have been thoroughly characterized and showed similar physico-chemical characteristics, indicating that the parboiling process does not introduce obvious lignin alterations. In addition, a preliminary evaluation of the potentiality of such lignin fractions as precursors of cement water reducers has provided encouraging results. A fermentation-based optional preprocess has also been investigated. However, both raw and parboiled RHs demonstrated a poor performance as a microbiological growth substrate, even in submerged fermentation using cellulose-degrading fungi. The described methodology appears to be a promising strategy for the valorization of these important waste biomasses coming from the rice industry towards a circular economy perspective.

## 1. Introduction

Rice (*Oriza sativa*) is one of the most important food crops, with about 755 million tons of rough rice (also known as paddy rice) produced worldwide in 2019 [1] and a >25% production increase in the 2000–2020 timeframe [2]. Its importance is undoubtedly established when it is taken into account that it feeds around three billion people all over the world.

Raw rice is composed by an external hull and the brown rice grain, which are separated during the dehusking process. Rice husks (or hull, RH), deriving from paddy rice dehusking, represent a very important source of waste biomass and are one of the most abundant by-products in the rice production line. Considering that ca. 20–25% of paddy rice mass is constituted by the outer husk [3], it is possible to estimate a global annual rice husk production of >150 million tons, currently used mainly for power generation [4].

Parboiled (partially boiled) rice is obtained from rough rice by a hydrothermal process constituted by a hydration stage followed by gelatinization and drying. Because of its several nutritional and physical benefits, parboiled rice possesses between two and three times the economic value of white rice and its importance on the global market is currently increasing (about 20% of the produced rice is treated by the parboiling process at the present time) [5,6]. The parboiling process is carried out mainly on rough rice that then must undergo the hulling process, thus leading to the parboiled rice husk as a by-product. As a consequence, this latter constitutes a significant part of the global rice husk production with solid forecasts of a high growth in the coming years.

For these reasons, the exploitation of both a raw rice husk (rRH) and parboiled rice husk (pRH) as lignocellulosic renewable feedstock constitutes a very important objective in a circular economy perspective, where the valorization of bio-agricultural wastes as a secondary raw material is largely preferable to their incineration for heat generation. Globally, this is particularly important in Asian countries where a rice cultivation is the primary food source and, regionally, in local realities where rice is a traditional source (e.g., in Lombardy in Italy). Up to now, RHs are instead mainly exploited as an energy source in rice processing plants and as bedding for farm animals. Therefore, the availability of this huge quantity of wastes and their low cost have pushed the current research towards the investigation and development of new sustainable possible applications for such materials.

Deep eutectic solvents (DESs) are a promising class of new solvents in the context of sustainable process development that can be prepared from a broad range of components, which are often from biobased sources [7]. DESs are characterized by a low toxicity and the typically high renewability and recyclability of the constituents [8]. Their wide solvating power modulation capabilities make them a first-choice medium for setting up separation processes, with particular reference to the fractionation of agro-industrial lignocellulosic wastes [9,10,11]. Moreover, it is even more interesting that the potential use of reactive DESs (RDESs, [12,13]) could allow for the exploitation of the same DES formulation as a separation medium (for lignocellulosic fractionation) and reaction medium (for lignin derivatization), even possibly in one-pot processes, enhancing the sustainability of the process [14].

**Figure 1:**
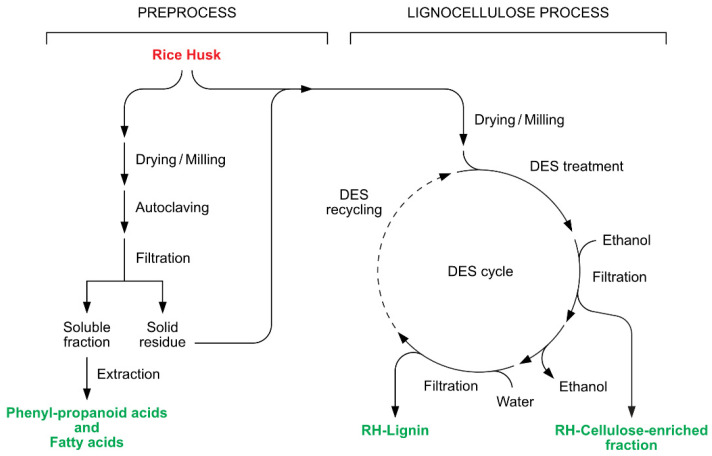
Complete fractionation process of raw and parboiled rice husks constituted by a hydro-thermal preprocess and a DES-mediated lignocellulose process.

Thus, we have focused on the set-up of a cascade multistep process, reported in Figure 1, composed of a preprocess in hot water and in an autoclave, followed by a DES-mediated treatment of the rRH and pRH biomasses, in order to deconstruct the natural lignocellulosic skeleton that usually hampers the further exploitation of the different components of RHs. The fractionation of these waste materials in two main fractions, i.e., a cellulose-enriched fraction and lignin constitutes the key-point for the successive development of valuable applications. The methodology based on the DES treatment was previously successfully applied by the authors to another common agri-food biomass, i.e., Brewer’s Spent Grain (BSG) [15]. The main aim of the present work is to study the DES-mediated fractionation process focusing on RHs, and to compare and characterize in detail both the composition and properties of the lignin fractions recovered from rRH and pRH. The latter is particularly interesting because of its aforementioned increase in commercial interest. Indeed, such a detailed characterization should represent a strategic milestone for the successful incorporation of lignin, as a macromolecular precursor, into bio-based polymers of a high added value [16,17,18], which have found an application in a variety of industrial [19,20,21,22] and technological [23,24,25,26,27,28] fields.

Moreover, in this work, the potentialities of these materials were also demonstrated in the field of cement water reducers, the latter being an important component of all modern concrete formulations. Lignin is a well-known component of traditional water reducers and has been widely used for this purpose for a long time [29]. In recent years, several research efforts aimed at the study of renewable high-performance water reducers inspired a renewed interest in advanced formulations based on lignin derivatives [30,31,32]. Any process resulting in lignin as a by-product is thus a potential resource for the development of sustainable concrete water reducers. For such reasons, the obtained lignins were here assessed for this specific application by rheological measurements on cement pastes. The results clearly indicate a water reducing performance equivalent to that of a commercial soda lignin (Protobind 1000), thus demonstrating the potential of the approach proposed herein.

A fermentation-based pretreatment was also studied, aiming to pretreat the biomass before the fractionation steps. The rationale behind this approach was to verify whether the soluble saccharides components of RHs could be exploitable as substrates in industrial fermentation processes. The expected effect of the pretreatment was the partial deconstruction of the lignocellulose matrix with the reduction in the saccharides’ content. However, the limited results obtained indicated that both raw and parboiled RHs are poor performers as a microbiological growth media, possibly due to their relatively low oligosaccharide and high silica contents.

## 2. Results and Discussion

In this work, two rice husk waste biomasses were selected from raw and parboiled rice production, respectively (see Section 3.1 for details). The present work aims to fractionate and compare them in terms of the composition and properties with the special aim of providing fundamental information for the exploitation of the different fractions, focusing in particular on the isolated lignins. Moreover, a deep comparison between rRH and pRH has not been presented until now.

**Figure 2:**
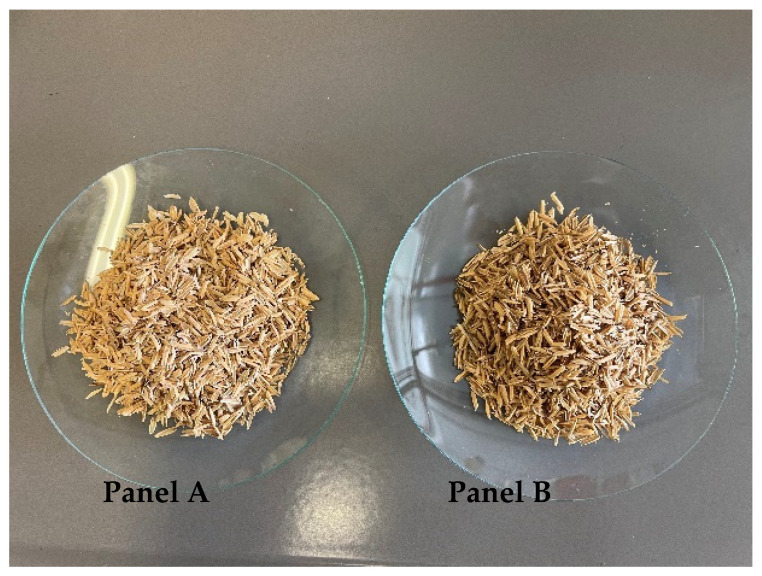
Picture of raw rice husk (**Panel A**) and parboiled rice husk (**Panel B**).

The two samples of the starting biomasses employed in the present work are illustrated in Figure 2. As it is evident from the picture, pRH (on the right) appeared to be slightly darker than rRH (on the left). As soon as they were received, these biomasses, even if they have been provided in a quite dried form, were put in a ventilated oven (60 °C for 24 h) and finely ground with an electric mixer.

### 2.1. Rice Husks Composition

As most lignocellulosic biomasses, RHs are an intricate material where all the components are closely related to each other. For that reason, in order to separate and quantify the four main constituents of rRH and pRH of this study (hemicellulose, cellulose, silica, and lignin), a multistep process based on a classical method of fractionation by successive water and acid treatments was performed (see Section 3.3 for details) [33]. The composition of the presently studied biomasses is reported in Table 1 and appears to be in agreement with those reported in the literature [34,35].

Moreover, the detailed composition, as monosaccharides components, of the isolated hemicellulose fraction (reported in Table 2) has been determined by GC-MS analysis after the total hydrolysis, reduction, and acetylation of the samples following a well-known procedure (see Section 3.3.1 for experimental details) [36]. The two main components of RHs hemicellulose were arabinose and xylose, with a low amount of galactose and traces of rhamnose and fucose. The detected glucose residues were probably due to a contamination of the cellulose fraction that appeared to be nearly completely eliminated after the pretreatment of the biomass performed in the fractionation process (the resulting residual glucose was 0.2–0.4%).

### 2.2. Rice Husks Preprocess

In our previous work, we described an efficient procedure for the water-mediated extraction of Brewer’s Spent Grain (BSG) [15]. Accordingly, the treatment of BSG with water at a high temperature allowed for the removal of a considerable (25–30% *w*/*w*) fraction of this waste material. Hence, we checked the effectiveness of the same procedure when applied to RHs.

#### 2.2.1. Hydro-Thermal Preprocess

We performed a hydro-thermal treatment of rRH and pRH by autoclaving the aqueous suspensions of the latter materials at 121 °C for 20 min. The two suspensions were then filtered, leading to solid residues which were indicated as rRH_T_ (raw rice husk-treated) and pRH_T_ (parboiled rice husk-treated), whereas the obtained aqueous extracts were concentrated at a reduced pressure and the weights of the resulting residues were compared to those of the parent RHs samples. We observed that the described procedure allowed for the extraction of only 2 and 2.8% (*w*/*w*) of rRH and of pRH, respectively. This water-mediated extraction gave results comparable with those described in Table 1, proving that rRH and pRH contained a very low amount of soluble sugars. In order to further characterize these soluble fractions, the extracts were further treated with ethyl acetate. The organic solvent soluble fractions were made up of a mixture of fatty acids (~0.05% *w*/*w* total biomass) and phenylpropanoid metabolites such as cinnamic acid (~0.74% *w*/*w* total biomass) and ferulic acid (0.13% *w*/*w* total biomass).

#### 2.2.2. Microbiological Preprocess

As discussed above, the results of the hydro-thermal preprocess have confirmed that both rRH and pRH are waste materials of a very difficult valorization, as they are devoid of water-soluble components such as sugars, starch, or proteins. In addition, the biomass of the RHs was almost unaffected by our thermal treatment. As a consequence, we evaluated whether a biological pre-treatment could break up the lignocellulosic structure of the RHs. To this end, we evaluated the potential of some selected fungal strains in the submerged fermentation of the RHs samples. More specifically, we singled out the filamentous fungi *Myceliophthora thermophila*, *Rhizomucor pusillus*, and *Trichoderma viride*. The latter microorganisms have been widely employed in the industry for the production of hydrolytic enzymes such as cellulases, amylases, pectinases, and chitinases [37,38,39,40]. In addition, the first two species are thermophiles with two different optimal growth temperatures. Therefore, the fermentation of the three identical samples of pRH with *M. thermophila*, *R. pusillus*, and *T. viride* were performed at 45, 35, and 24 °C, respectively. The pRH biomasses recovered after the fermentation indicated a weight loss of the 18, 19, and 14%, respectively. These results clearly demonstrate that all the tested strains utilized pRH as a carbon source for their growth, regardless of the temperature fermentation. Despite this fact, none of the experiments displayed a weight loss superior to 19%, thus confirming the difficult degradability of RHs, even when using cellulose-degrading fungi. Interestingly, for all the trials, we observed an initial luxuriant growth followed by the transformation of the formed filamentous biomass into a slurry. The microscopic view of the biomasses showed the presence of a very short hypha, most likely deriving by the grinding of the fungal filaments. We supposed that RH, with its high silica content, was not a suitable substrate for a submerged fermentation. The shaking, necessary for oxygenation, damaged the fungal hypha, thus reducing their activity. As a consequence of the described results, the microbiological preprocessing of this specific waste was considered to be unsuitable and thus excluded from the complete fractionation process depicted in Figure 1.

### 2.3. DES-Mediated Lignocellulose Process

The subsequent step of the biomass treatment after the previously described preprocess was constituted of a DES-mediated fractionation process. Different DESs have been prepared and tested in this work. They have been obtained by mixing and heating a mixture of the two components: a hydrogen bond acceptor (HBA) such as choline chloride (ChCl) and betaine glycine (BetG), and a hydrogen bond donor (HBD) composed by acetic acid and L-lactic acid. These two HBDs have been selected since it is known that the presence of the carboxylic group in an HBD enhances the lignin yields during the lignocellulosic destructuration [41,42]. The composition and density of the DESs are reported in Table 3. Typically, DESs had a higher density than water and those containing L-lactic acid (DESs 2 and 4) resulted in being denser than those with acetic acid (DESs 1 and 3), likely because of the presence of the hydroxyl group in the HBD molecules, which should allow for the formation of stronger hydrogen bonds.

**Figure 3:**
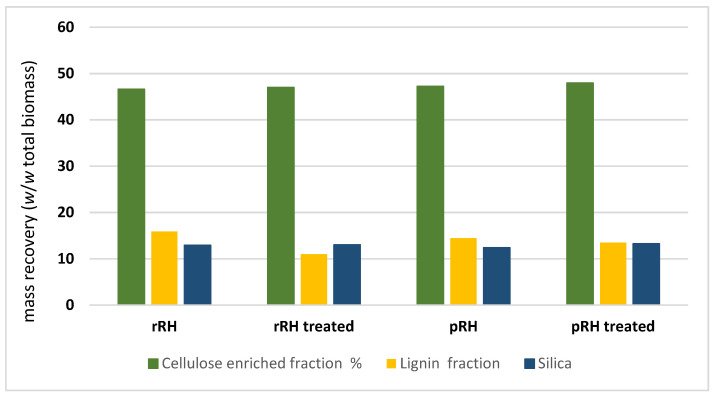
Data of mass recovery of cellulose-enriched fraction (in green), lignin (in yellow), and silica (in blue) during the DES 2-mediated fractionation process of raw rice husk (rRH), raw treated rice husk, parboiled rice husk (pRH), and parboiled treated rice husk.

Following the scheme of the process reported in Figure 1, dried and finely milled treated biomasses (rRH_T_ and pRH_T_) were submitted to a fractionation. For comparison, the native biomasses (rRH and pRH) were also treated in the same way. The biomasses were then suspended in the different DESs at 120 °C under magnetic stirring for 24 h. After cooling, ethanol was gradually added, leading to the precipitation of the first fractions, the cellulose-enriched fractions, which were then further separated in order to quantify the silica content. To this end, we slightly modified the Yoshida procedure [43], consisting in the oxidative degradation of all the organic fractions (see Section 3.5.3 for details). The cellulose-enriched fractions were heated at a reflux with a mixture of concentrated nitric acid, sulfuric acid, and perchloric acid, and the resulting silica suspensions allowed for the isolation of the silica samples. Their weights were employed to calculate the silica content of the cellulose-enriched fractions (given as a *w*/*w* biomass percentage and reported in Figure 3). The silica content was quantified at around 13%, almost identical in all the samples, confirming that the treatments performed on the RHs did not affect their SiO_2_ content.

The filtrates were then concentrated to eliminate ethanol and were treated with water acting as an anti-solvent, to induce the precipitation of the lignins. After the centrifugation, filtration, and solvent evaporation, the final solid fractions (rRH-Lignin, rRH_T_-Lignin, pRH-Lignin, and pRH_T_-Lignin) were recovered and quantified.

After the examination of the results obtained with the four DESs in terms of the handling of the reaction mixtures and the final mass recovery, DES 2 (choline chloride/L-lactic acid, 1/5) was selected for this work, since it gave the higher yields in terms of the fractions recovery and lignin recovery. The data of the quantitative recovery of the different fractions are reported in the Supplementary Materials (Table S1) for all the tested DESs, whereas the mass recovery results obtained on the DES 2-mediated fractions for the four RHs biomasses have been illustrated more precisely in Figure 3.

As it appears clearly from Figure 3, the weight percentages of the three fractions were almost identical for the four fractionated RHs. The cellulose-enriched fraction accounted for 47% of the total biomass in the three cases, lignin for 15.8 to 11% (for rRH and rRH_T_) and to 14.5 to 15.5% (for pRH and pRH_T_), and silica at around 13% in all cases. The results clearly prove that both the parboiling process and the autoclave treatment do not influence so much the percentages of the three main fractions and do not induce substantial differences in the natural biomass fractionation.

### 2.4. Lignin Characterization

Lignins derived from the complete fractionation process (using DES 2) of both raw and parboiled rice husks (rRH-Lignin and pRH-Lignin, respectively) were subjected to an extensive characterization to evaluate their properties and potential differences. A well-known commercial soda lignin (Protobind 1000, indicated from now on as Protobind) was used as the reference [44].

#### 2.4.1. Solvent Solubilization

**Figure 4:**
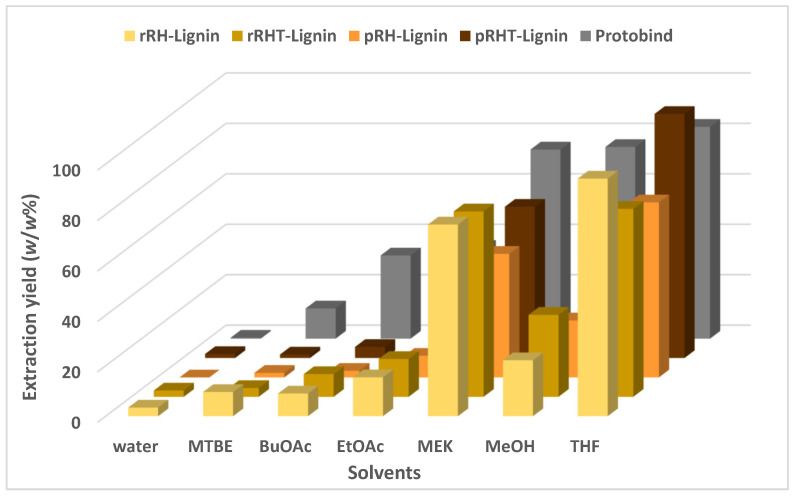
Extraction yield of the commercial technical lignin Protobind (in grey), and the lignins extracted from raw (in pale yellow), raw treated (dark yellow), parboiled (in orange), and parboiled treated (in brown) rice husks. The data are reported as the percentage (*w*/*w*) of solubilized fraction vs. total fraction in 6 different organic solvents and water.

One of the key features in the lignin’s characterization and valorization is represented by the solubilization studies, which can afford the starting point in the set-up of the best conditions for lignin processing. In fact, it should be stressed that one of the main issues concerning lignin exploitation is its low solubility in the most frequently used organic solvents. In this work, the solubilization screening has been performed in six organic solvents and in water. The selected solvents have been: *tert*-butyl methyl ether (MTBE), *n*-butyl acetate (BuOAc), ethyl acetate (EtOAc), 2-butanone (MEK), methanol (MeOH), and tetrahydrofuran (THF). Figure 4 shows the obtained extraction yields for rRH- (in pale yellow), rRH_T_- (in dark yellow), pRH- (in orange), pRH_T_-Lignins (in brown), and Protobind (in grey) for comparison, as a *w*/*w* percentage of the solubilized lignin vs. total lignin.

As expected, the four RH-lignins are quite insoluble in water, similar to what was observed in the reference material (Protobind). When considering the solubility response to organic solvents, RH-lignins show a substantial similarity in their solubility: a very poor solubility (less than 10% *w*/*w*) in MTBE and BuOAc, and a slightly higher solubility in EtOAc and MeOH in a range between 10 and 30% *w*/*w*. The best solvents appear to be MEK and THF, as for the reference material (Protobind), where the value of the solubility ranges from 50 up to 99% *w*/*w*. It should be noted that the lignins from rRHs seemed to be slightly more soluble in all the solvents, except THF, if compared with the pRHs. Moreover, the solubility of the RH_T_-lignins appeared to be higher than the RH-lignins in all the organic solvents (except THF), indicating that the biomass pretreatment could generally positively affect the solubility characteristic of the recovered lignins. This aspect appears as an interesting outcome and suggests a potential straightforward exploitation of such RH-recovered lignins in line with the similar exploitation routes typical of commercial lignin materials.

#### 2.4.2. Molar Mass Distribution

The evaluation of the molar mass distribution in the lignin recovered fractions is a fundamental tool in order to successfully exploit these biomasses. To that end, GPC analyses were performed to determine the molecular weight and the molecular weight distribution of the extracted lignins. In Table 4, the number average molecular weight (M_n_), the weight average molecular weight (M_w_), and the polydispersity index (*Ð*) of all the RH-lignin samples and of the reference lignin Protobind 1000 are reported.

The RH-Lignins showed a higher M_n_ and M_w_ and a similar *Ð*, with respect to the Protobind lignin. Especially, in all RH-Lignins, a comparable M_n_ was observed. In contrast to this, the M_w_ of both rRH-Lignins was found to be higher compared to both pRH-Lignins, resulting in a higher *Ð*. No differences were observed between the treated and untreated lignins, indicating that the pretreatment did not affect the molecular weight and the molecular weight distribution of these lignins.

These results are in accordance with what was previously discussed in terms of solubility. Indeed, on average, due to their higher M_n_ and M_w_, RHs-lignins resulted in being slightly less soluble in organic solvents compared to the Protobind lignin.

#### 2.4.3. Sugar Content

The precipitated lignin fractions were washed three times after the water-mediated precipitation from the DES solutions in order to eliminate potential DES residues, which could contaminate the lignins and provide a sticky response of the final products. The sugar quantification, reported in Table 5, was performed with an already established procedure using the bicinchoninic assay method with some modifications [15,45]. Although they were not further purified after the precipitation, all the lignins possessed a very low carbohydrate content similar to the Protobind value; it accounted for less than 0.4% (*w*/*w*) of the free reducing sugars. Furthermore, after the hydrolysis, the rRH- and pRH-Lignins showed the presence of a certain quantity of complex sugars (from 5.7 to 9.1%), which was also in this case substantially lower than the Protobind value (13%). However, pRH_T_-Lignin exhibited quite the same value as Protobind. This aspect has to be considered in view of the further applications of these RH-Lignins in the preparation of cement water reducers.

#### 2.4.4. ^13^C CP-MAS NMR

**Figure 5:**
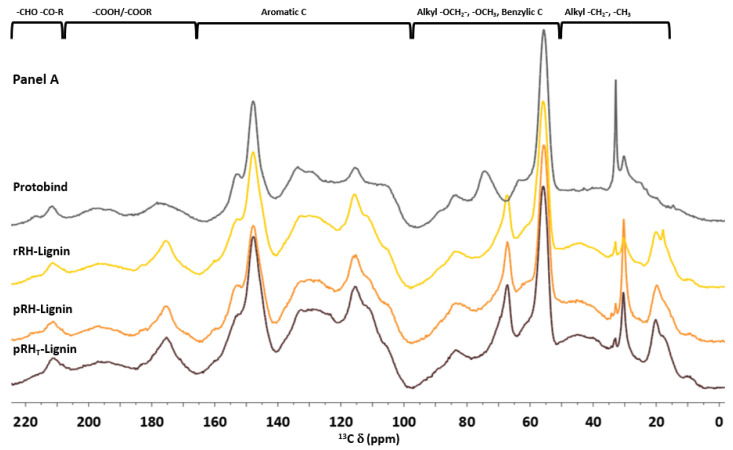

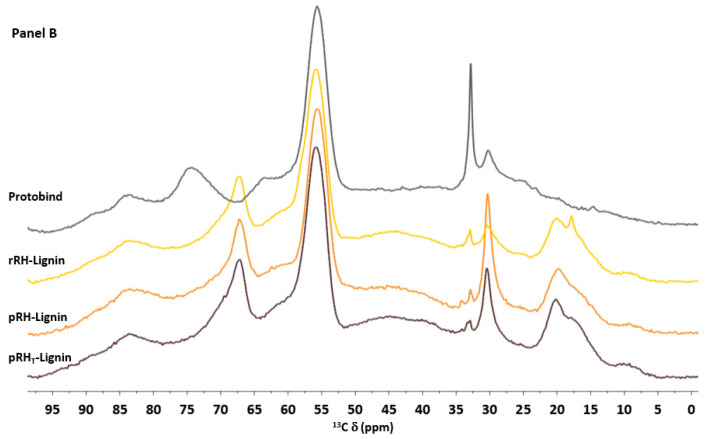
Solid state ^13^C CP-MAS NMR spectra of Protobind lignin (in grey), rRH-Lignin (in yellow), pRH-Lignin (in orange), pRH_T_-Lignin (in brown). (**Panel A**) the whole spectrum; (**Panel B**) zoom of 0–100 ppm region.

The solid-state ^13^C cross-polarization magic angle spinning (CP-MAS) NMR spectroscopy was used to define the fingerprint of each lignin and to highlight the structural differences among the examined samples. Figure 5 shows the superposition of the ^13^C CP-MAS NMR spectra of the commercial Protobind lignin with the extracted lignin samples. At first, the RHs samples had an almost overlapping profile but clearly differed from the Protobind reference sample (see Figure 5 Panel A). The region between 210 and 190 ppm corresponded to the non-conjugated carbonyl groups C=O of aldehydes. In this range of chemical shifts, there were no substantial differences among the spectra, whereas in the aromatic region (160–100 ppm), Protobind showed a lower signal intensity at 175.7 ppm compared to the other spectra. This indicated a lower presence of both –CO_2_H carboxylic and –CO_2_R ester groups. In addition, the Protobind spectrum showed another signal which was less intense than the other samples around 115 ppm, which very likely could be attributed to a lower content of the guaiacyl units.

However, the main spectral differences were between 100 and 0 ppm (Figure 5 in Panel B). Between 65 and 75, Protobind presented two more peaks at 74.5 and 63.7 ppm: peaks in such a region are typical of the β-O-4 structural moiety with the –OH group at the α-position. On the other hand, the extracted lignin samples showed at 67.4 ppm a signal which was not present in the Protobind lignin. This region (62–75 ppm) is diagnostic of β-O-4 structures. In the alkyl part of the spectra, the intensity of the O-CH_3_ peak at 55.9 ppm was comparable in all the samples. Lastly, the most significant differences were at around 30 ppm (corresponding to the alkyl CH_2_) and around 20 ppm (typical of the methyl groups). This analysis could be a very important tool in order to compare the lignins extracted from different biomasses.

#### 2.4.5. Total Phenolic Content

The total phenolic content was determined in the recovered lignins from RHs using a modified Folin–Ciocalteu (FC) assay (see Section 3.6.5 for details). In this protocol, the samples were initially fully solubilized in DMSO before being incubated with the specific redox reagent (FC reagent). The phenolic content results are summarized in Table 6 as the vanillin equivalents (mmol/g of dry lignin), using the data from Protobind as the reference.

All four lignins from RHs show quite the same content of vanillin equivalents (around 1.3–1.6 mmol/g), which is half the value found in Protobind. In addition, also in this case it appears that the parboiled treatment does not affect the majority of the functionalities of the phenolic lignin.

#### 2.4.6. Hydroxyl Groups Quantification by ^31^P NMR

**Figure 6:**
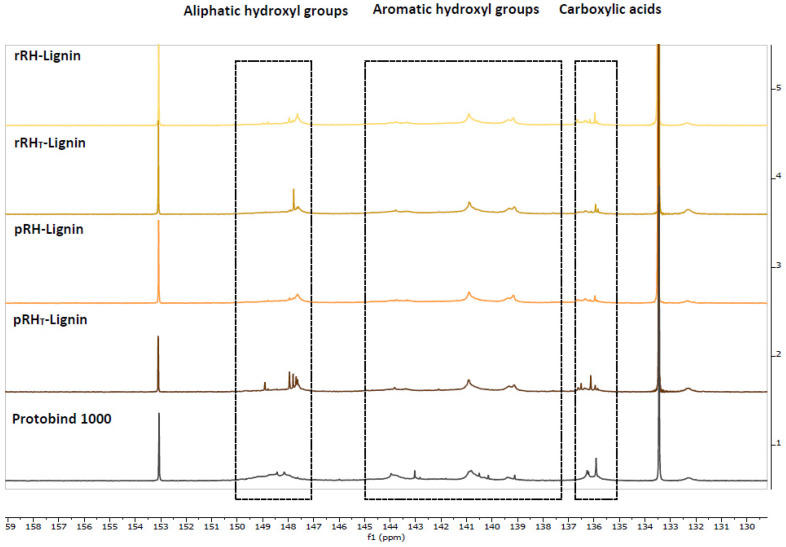
^31^P NMR Spectra of isolated Lignins and Protobind as reference.

The different hydroxyl groups present in the recovered lignins were assessed and identified through high resolution ^31^P NMR spectroscopy. This technique allows for the quantification of the different hydroxyl groups present in the lignin backbone after derivatization with 2-chloro-4,4,5,5-tetramethyl-1,3,2-dioxaphospholane [46,47]. The high natural abundance of the ^31^P nucleus allows us to obtain well-resolved NMR signals. Their attribution to the different OH groups in the sample could be performed because the chemical shifts are largely dependent on the surrounding chemical environment of the derivatized hydroxyls. The spectra are reported in Figure 6.

The signals of the RH-Lignins and Protobind spectra were integrated to differentiate the hydroxyl groups: the signals from 150 to 147 ppm were associated with the aliphatic hydroxyl groups, the signals from 145 to 138 ppm were related to the aromatic hydroxyl groups, and, lastly, the signals centered at 136 ppm were attributed to the carboxylic acid residues. The peak integration of these three chemical shift regions led to the quantification of the total hydroxyl groups expressed in mmol of the functional group per g of dry lignin, as reported in Table 7 below.

Additionally, in this case, the ^31^P NMR results showed that the three RH-Lignins had quite the same amount of hydroxyl and carboxyl groups in their composition. In particular, when compared with Protobind, the lignins extracted from RHs appeared to have quite the same quantity of aliphatic OH and carboxylic groups, whereas the main differences were showed in the aromatic hydroxyl groups, which appeared to be one and a half times higher than in Protobind.

#### 2.4.7. Fourier-Transform Infrared Spectroscopy

**Figure 7:**
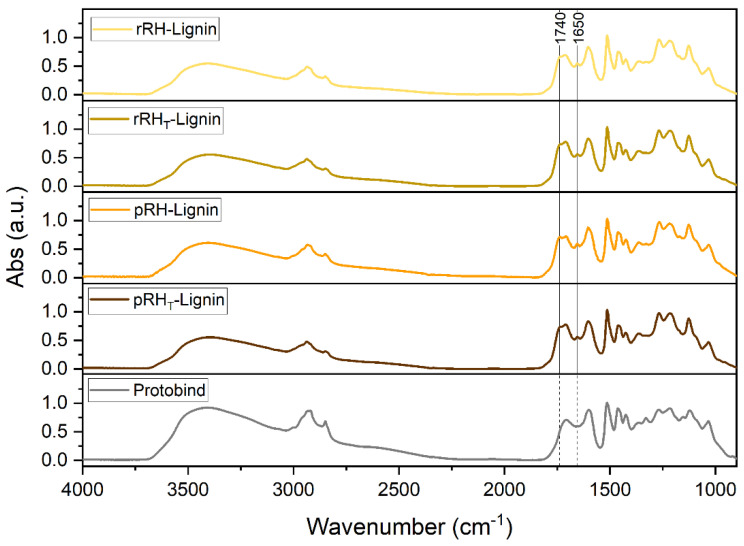
FT-IR spectra of rRH-Lignin (in pale yellow), rRH_T_-Lignin (in dark yellow), pRH-Lignin (in orange), pRH_T_-Lignin (in brown), and Protobind (in grey).

Fourier-transform infrared (FTIR) spectroscopy was carried out to further study the chemical composition of the RH-Lignins. The absorption spectra obtained are shown in Figure 7, where the FTIR spectrum of Protobind is also presented for comparison purposes. In general, no noticeable differences among the recovered lignins could be observed. All the lignin fractions showed a broad absorption band in the region of 3400–3200 cm^−1^, related to the stretching vibrations of the aliphatic and phenolic OH groups, and the signals in the region of 3050–2800 cm^−1^ were associated with the CH bond stretching in the methyl and methylene groups. The major differences between the RH-Lignins and Protobind lignin were found in the fingerprint region. In particular, while Protobind showed a single signal located at 1710 cm^−1^ attributable to the stretching vibration of C=O in unconjugated ketones, carbonyl, and ester groups, all four RH-Lignins were characterized by an additional peak at 1740 cm^−1^. This signal was associated with C=O stretching vibrations likely resulting from the presence of different carbohydrate species entrapped in the recovered RH-Lignin fractions, as also evident from Table 5. This result was also confirmed by the signal detectable at 1650 cm^−1^ for such materials, related to conjugated carbonyl/carboxyl stretching in C=O moieties [48].

#### 2.4.8. Thermal Behavior

**Figure 8:**
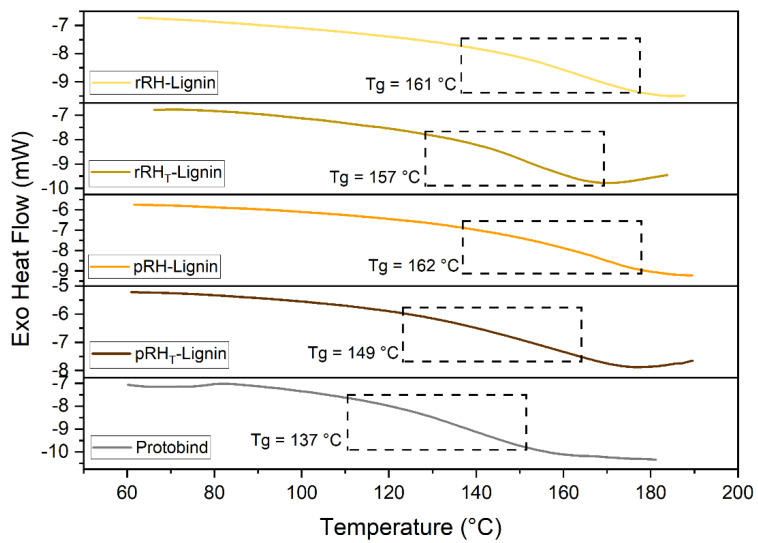
DSC scans (second heating ramp) of rRH-Lignin (in pale yellow), rRH_T_-Lignin (in dark yellow), pRH-Lignin (in orange), pRH_T_-Lignin (in brown), and Protobind (in grey).

The glass transition temperatures (Tg) of all the lignin samples were assessed by means of differential scanning calorimetry (DSC). The thermograms reported in Figure 8 showed that the Tg of the recovered RH-Lignins were slightly higher compared to that of the reference lignin material (Protobind). In particular, rRH- and pRH-Lignins were characterized by the same Tg, close to 160 °C. This value was higher compared to the Tg of the rRH_T_- and pRH_T_-Lignin, which instead were detected at around 157 and 149 °C, respectively, meaning that the treatment has slightly affected the molecular structure and thus the macromolecular mobility of such a lignin fraction. The behavior is perfectly in accordance with the results of both GPC analyses and ^31^P NMR. Especially, the GPC analyses showed a higher Mw and Mn of the RH-Lignins. Along the same lines, ^31^P NMR allowed us to detect a higher abundance of OH aromatic functionalities, which can induce the higher intra/intermolecular hydrogen bonding capability, ultimately resulting in a reduced molecular motion (viz., reduced free volume and higher Tg).

**Figure 9:**
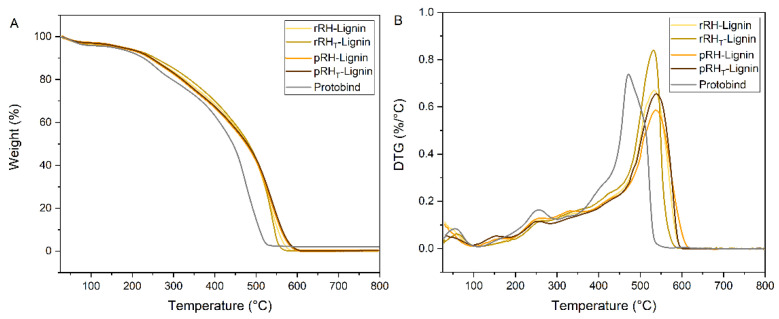
(**A**) TGA and (**B**) DTG traces of rRH-Lignin (in pale yellow), rRH_T_-Lignin (in dark yellow), pRH-Lignin (in orange), pRH_T_-Lignin (in brown), and Protobind (in grey). Analyses were conducted in air.

To study the thermal stability behavior of the lignin samples, thermogravimetric analysis (TGA) was performed as well. The mass loss traces as a function of the temperature of the RH-Lignins and of Protobind are reported in Figure 9, where the mass derivative (DTG) is also shown.

As it can be observed, all RH-Lignin fractions exhibited a slightly improved thermal response as compared with the reference material (Protobind) over the entire temperature range which was investigated. This can be associated with the slightly higher molecular weights (and characteristic thermal transitions) found in RH-Lignins, which result in a higher stability towards thermo-oxidative degradation. In particular, a relatively monotonic mass loss response is observed, where a single broad degradation event can be detected. This behavior is reflected in the temperatures at which 10% and 50% mass losses are registered, which appear in line with Protobind (Table 8).

### 2.5. Water Reduction in Cement Pastes

**Figure 10:**
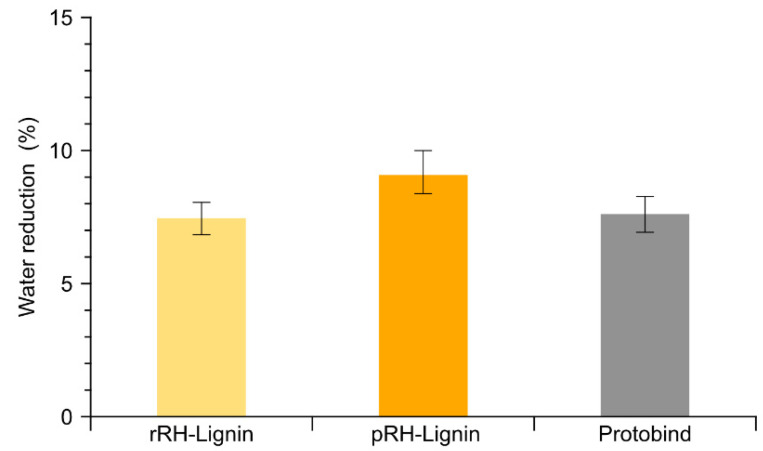
Measured water reduction capability for cement pastes containing 0.2% wt. of lignin. Error bars are the confidence interval (1σ) of the inverse linear regression.

Natural lignins are a very interesting resource for the development of sustainable traditional cement water reducers. Moreover, high-performance lignin-based cement water reducers can also be developed through different types of lignin derivatization [49,50,51,52]. Typically, lignosulfonates show better water-reducing capabilities than natural lignins, but even the latter have been successfully adopted as a starting point in the development of high-performance experimental cement plasticizers. This was obtained both in the presence [53] and in the absence of sulfonation steps [49]. The DES-mediated fractionation of the lignocellulosic biomass is a mild process that results in the production of non-derivatized lignins comparable to those obtained by organosolv or soda processes. The lignins (rRH- and pRH-Lignins) obtained in the present study were thus evaluated using a commercial soda lignin (Protobind) as a reference. The measured water reduction capabilities, made by cement pastes (a blend of water and cement powder), are reported in Figure 10.

The results indicated a comparable water reduction activity for the lignins derived from the rRH and pRH samples. Both lignins demonstrated an equivalent performance in comparison to the commercial soda lignin Protobind. The reported data are in a good agreement with a previous study based on BSG as a lignocellulosic starting material [15]. Indeed, the obtained results indicate that the lignin derived from the proposed process is comparable to a typical industrial soda lignin and that it constitutes a suitable basis for the development of sustainable, high performance concrete water reducers. Moreover, these results indicate that the lignin derived from the pRH is comparable to that of rRH.

## 3. Materials and Methods

### 3.1. Materials and General Methods

The rice husk samples (from *Oriza sativa*) were kindly provided by Riso Scotti S.p.A. (Pavia, Italy) as raw rice husk derived from the processing of *japonica* (90%) and *indica* (10%) rice varieties and parboiled rice husk derived from *indica* (75%) and *japonica* (25%) varieties. Protobind 1000 (a mixed wheat straw/sarkanda grass lignin from the soda pulping of non-woody biomass) was provided by Tanovis (Alpnach, Switzerland).

All air- and moisture-sensitive reactions were carried out using dry solvents and under a static atmosphere of nitrogen. Choline chloride (C0329), betaine glycine (B0455) and L-lactic acid (L0165), D-(+)-Galactose, and L-(−)-fucose were purchased from TCI (Milano, Italy), whereas (L)-(+)-rhamnose, L-(+)-arabinose, D-(+)-xylose, D-(+)-mannose, D-(+)-glucose, and the other reagents and the employed solvents, used without a further purification, were obtained from Merck (Merck Life Science S.R.L., Milan, Italy).

Thin layer chromatography (TLC) Merck silica gel 60 F_254_ plates (Merck Millipore, Milan, Italy) were used for analytical TLC.

The identification and the quantification of the low molecular weight aromatic compounds obtained in the pre-treatment step were carried out using gas chromatography coupled with mass spectrometry (GC-MS). The GC-MS apparatus used is an Agilent GC System 7890A, with an inert MSD with a Triple-Axis Detector 7975C. The gas carrier was helium at a flux of 1.18 mL/min. The separation was performed on a DB-5MS column (30 m × 250 μm × 0.25 μm, Phenomenex, Bologna, Italy) with a temperature program of 50 °C (1 min) to 280 °C at 10 °C/min, 280 °C at 15 min (total run time 39 min). A solvent delay of 4 min was selected. The samples were dissolved in methanol or acetone in a concentration of around 0.5–1 mg/mL.

### 3.2. Microorganisms and Growth Media

*Myceliophthora thermophila* (CBS 866.85) and *Rhizomucor pusillus* (CBS 354.68) were purchased from the CBS-KNAW collection (Utrecht, The Netherlands). *Trichoderma viride* (DSM 63065) was purchased from the DSMZ GmbH collection (Braunschweig, Germany).

The microbial inoculum for the RH pretreatment consisted of the suspension of the suitable fungal spores in distilled water. The latter suspensions were prepared adding sterile water to the suitable fungal culture, grown on an agar slant, followed by the scrubbing of the sporulated surface.

Trace elements solution: FeCl_3_ (50 mM), CaCl_2_ (20 mM), MnCl_2_ (10 mM), ZnSO_4_ (10 mM), CoCl_2_ (2 mM), CuCl_2_ (2 mM), NiCl_2_ (2 mM), Na_2_MoO_4_ (2 mM), Na_2_SeO_3_ (2 mM), and H_3_BO_3_ (2 mM). All inorganic salts were purchased from Carlo Erba (Milano, Italy).

### 3.3. Determination of Rice Husks Composition

The rRH and pRH compositions have been determined with a known multistep procedure with minor modifications [33]. The RH (1 g, value a) was suspended and stirred in deionized water (150 mL) at 100 °C for 1 h. Then, after filtration, the solid was washed by deionized water (300 mL), dried in an oven at 80 °C, and weighted (value b). The solid was treated with 1 N of H_2_SO_4_ (150 mL) at 100 °C for 1 h. Then, the suspension was filtered again, washed with water, and the solid was dried and weighted (value c). The solid was mixed with 72% H_2_SO_4_ (10 mL) for 4 h at r.t. and treated with 1 N of H_2_SO_4_ (150 mL) under a reflux for 1 h. After cooling, the solid was isolated by filtration, dried, and weighted (value d). The final residue was then calcinated in an oven at 600 °C for 6 h and the residue has been quantified (value e). The fractions of the different components were quantified with the following equations:

hemicellulose (%) = 100 × [(b − c)/a];

cellulose (%) = 100 × [(c − d)/a];

lignin (%) = 100 × [(d − e)/a].

#### 3.3.1. Determination and Quantification of Hemicellulose Components

The procedure was performed according to Foster et al., with minor modifications [36]. The monosaccharides fraction obtained from the hemicellulose hydrolysis (obtained in Section 3.3) was dissolved in deionized water (30 mL) and was treated with NaBH_4_ (1 g, 26.4 mmol) stirring at r.t. for 2 h. Then, the reaction was quenched by the careful addition of glacial acetic acid (10 mL), keeping the temperature under 30 °C by external cooling. The solvent (water) and the excess of acetic acid were removed by evaporation under a reduced pressure and the obtained powder was treated with pyridine (30 mL) and acetic anhydride (30 mL) stirring at a reflux for 1 h. Hence, the reaction was concentrated to the dryness under a reduced pressure and the residue was partitioned between ethyl acetate (70 mL) and water (100 mL). The aqueous phase was extracted again with ethyl acetate (50 mL) and the combined organic phases were washed in turn with saturated NaHCO_3_ aq. (100 mL) and with brine (100 mL). The resulting solution was dried (Na_2_SO_4_) and concentrated in vacuo. The residue contained the alditol acetates of the hemicellulose monosaccharides, whose relative composition was determined by GC-MS analysis. GC-MS analyses for the determination of sugar composition in hemicellulose were performed on an HP-6890 gas chromatograph equipped with a 5973 mass detector and using an HP-5MS column (30 m × 0.25 mm, 0.25 µm film thickness; Hewlett Packard, Palo Alto, CA, USA). The temperature program was the following: 120 °C (3 min)—12 °C/min—195 °C (10 min)—12 °C/min—300 °C (10 min); carrier gas: He; constant flow 1 mL/min; and split ratio: 1/30. The reference standards of the alditole acetates were prepared starting from the corresponding monosaccharides, following the reduction/acetylation protocol described above. The retention times of the alditole acetates are given below for each monosaccharide derivative: rhamnose 14.03 min; fucose 14.30 min; arabinose 14.46 min; xylose 14.88 min; mannose 21.55 min; glucose 21.65 min; and galactose 21.96 min.

### 3.4. Rice Husks Preprocess

#### 3.4.1. Hydro-Thermal Preprocess

A sample of rice husk (30 g) was suspended in distilled water (1 L) and was heated in an autoclave at 121 °C for 20 min. After cooling, the solid residue was removed by filtration and the aqueous phase was concentrated under a reduced pressure until it reached complete dryness. The crude extracts of rRH and pRH were 2.1% and 2.8% *w*/*w* of the starting biomass, respectively. They were further fractionated by extraction with ethyl acetate and filtrated on a short silica gel column. The so-obtained solvent soluble fractions were derivatized as previously described and then analyzed by GC-MS [54]. Briefly, a mixture of 25 μL of pyridine, 250 μL of dioxane, and 75 μL of silylation mixture composed of N,O-bis(trimethylsilyl)trifluoroacetamide (BSTFA, Sigma T6381) with trimethylchlorosilane (TMC, Sigma T6381) was incubated with 1 mg of the sample heated in a thermomixer (1.5 mL vial Eppendorf Thermomixer Comfort) at 70 °C and 600 rpm for 30 min. At the end, 100 μL of the mixture were withdrawn, added to 100 μL of dioxane, and analysed by GC-MS with the same apparatus and analytical method reported in Section 3.1. The identification of the compounds was performed by means of an NIST 2008 mass spectral library search and then the selected peaks were confirmed with the known standards (comparing both the mass spectrum and chromatographic coordinate).

#### 3.4.2. Microbiological Preprocess

A sample of ground pRH (25 g) was added to a 1 L flask which contained distilled water (250 mL), yeast extract (0.3 g), NH_4_Cl (0.8 g), and the microelement solution (4 mL). The flask was sealed with a cellulose plug and was sterilized at 121 °C for 15 min. Then, the obtained mixture was inoculated with the selected strain and the microbial growth was performed according to the experimental conditions indicated below.

*Myceliophthora thermophila* (CBS 866.85): 45 °C, 6 days, 130 rpm

*Rhizomucor pusillus* (CBS 354.68): 35 °C, 8 days, 130 rpm

*Trichoderma viride* (DSM 63065): 24 °C, 8 days, 130 rpm

Hence, the microbial transformation was interrupted by filtration on a narrow mesh metallic filter. The recovered RH was washed with deionized water and was dried (ventilated oven, 60 °C, 24 h).

According to this procedure, the difference between the weight of the recovered pRH and the weight of the untreated sample takes into account of the sum of the husk soluble fractions and of the husk organic fraction depleted by the fungal strain during its growth process. Overall, the fraction between this weight difference over the corresponding RH initial weight (given as *w*/*w* percentages) indicates the degradative capability of the strain. The obtained results are listed below:

*Myceliophthora thermophila*: 18%; *Rhizomucor pusillus*: 19%; and *Trichoderma viride*: 14%.

### 3.5. Rice Husks Lignocellulose Process

#### 3.5.1. Preparation of DESs

The DESs were prepared in a closed flask by mixing the anhydrous hydrogen bond acceptor (HBA) with the hydrogen bond donor (HBD) in the determined molar ratio (see Section 2.3) and was stirred at 120 °C for 4 h until the liquid phase appeared to be completely homogeneous and clear. The products were then dried under a vacuum and stored at room temperature in a desiccator in the presence of anhydrous calcium chloride until further use. The ^1^H NMR spectrum of the selected DES 2 (choline chloride/L-lactic acid 1/5) for the full fractionation is reported in the Supplementary Materials (Figure S1).

#### 3.5.2. DES-Mediated Lignocellulose Process

The rice husk (25 g) was suspended in a DES of choline chloride/L-lactic acid 1/5 (250 mL) at 130 °C in a round-bottom flask under magnetic stirring for 24 h. After cooling, ethanol (500 mL) was then added gradually over 2 h in order to precipitate the cellulose-enriched fraction. The solid particulate was separated by centrifugation and filtration, washed many times with ethanol, and dried to give a final solid RH-Cellulose-enriched fraction (~15 g) with a yield of 59–61% (*w*/*w* initial biomass). This fraction was further fractionated in order to quantify the silica content (see Section 3.5.3). The silica content accounted for 12.8% (*w*/*w* biomass).

The filtrate after the ethanol treatment was then concentrated by the rotary evaporation under the vacuum to eliminate the solvent. Water (500 mL) was added, and the suspension was stirred for 24 h at 4 °C. The obtained precipitate was then centrifuged, filtered, and washed three times for 1 h with a solution of water/ethanol 9/1. After the centrifugation, filtration, and solvent evaporation, the final fraction (RH-Lignin, ~3 g) was recovered with a final yield of 10% (*w*/*w* initial biomass).

#### 3.5.3. Determination of Silica Content in Cellulose-Enriched Fractions

The cellulose-enriched fractions, derived from DES-mediated fractionation, were analyzed in order to determine their silica contents, using the Yoshida procedure based on the complete oxidation of the organic materials by heating with a mixture of strong acids with some modifications. We employed an acid mixture with the following composition: HNO_3_ aq. (65% *w*/*v*) 75 mL, H_2_SO_4_ aq. (96% *w*/*v*) 15 mL, and HClO_4_ aq. (70% *w*/*v*) 30 mL.

Each sample of the cellulose-enriched fractions (about 2 g, dry weight) was placed in a round bottomed flask containing a stirring bar and equipped with a condenser. The fractions were treated with 20 mL of the acid mixture and were allowed to predigest under a fume hood for 3 h. Then, the obtained brown slurries were heated at reflux under stirring for six h. The resulting colorless silica suspensions were ice-cooled, diluted with 60 mL of distilled water, and centrifuged (9000 rpm, 4 °C). The collected silica samples were washed with distilled water (2 × 25 mL), with acetone (25 mL), and were then dried at a reduced pressure. The weights of the obtained silica samples were employed to calculate the silica content of the cellulose-enriched fractions and thus of the starting rice husk samples (both given as a *w*/*w* percentage).

### 3.6. Lignin Characterization

#### 3.6.1. Solvent Solubilization Determination

The lignin solvent’s solubility was determined by treating 1 g of the analyzed lignin with 10 mL of the different solvents under stirring at 400 rpm. Each test was carried out overnight at room temperature. The suspension was then filtered and the solvent was evaporated at a reduced pressure, and the final residue was dried until a constant weight was achieved prior to the quantification.

#### 3.6.2. Molar Mass Determination

A Waters 510 HPLC system equipped with a refractive index detector was used for the GPC analyses. Tetrahydrofuran (THF) was used as the eluent. The analyzed lignin sample (volume 200 μL, concentration 1 mg/mL in THF) was injected into a system of the three columns connected in series (Ultrastyragel HR, Waters − dimensions 7.8 mm × 300 mm) and the analysis was performed at 30 °C at a flow rate of 0.5 mL/min. The calibration was performed against polystyrene standards in the 10^2^−10^4^ g/mol molecular weight range. The samples have been acetylated to allow for a complete solubility in the THF eluent. The estimation of the number-average and weight-average molecular weights of the obtained lignin fractions was performed excluding the signals related to the solvent (THF) and the solvent stabilizer (butylated hydroxytoluene), visible at long elution times (>29.5 min).

#### 3.6.3. Sugar Content Determination

The total sugars quantification was performed with a previously described method based on a bicinchoninic assay [15,44].

#### 3.6.4. ^13^C CP-MAS NMR Analysis

Solid state NMR experiments were carried out on a Bruker NEO 500 MHz spectrometer (Bruker, Billerica, MA, USA) equipped with an i-probe solid state probe. The samples (150 mg) were packed into 4 mm zirconia rotors and sealed with Kel-F caps. All the NMR spectra were recorded at 298 K. The ^13^C CP-MAS NMR spectra were performed at 125 MHz. The spin-rate was kept at 8 KHz. The 90-pulse width was 3.2 μs, the relaxation delay was 4 s, the acquisition number was 17,000, and the contact time was 1.5 ms. The spectra were obtained using 1024 data points in the time domain, zero-filled, and Fourier transformed. All the data were processed using the MestreNova 6.0.2 software (Mestrelab Research, Santiago de Compostela, Spain). An adamantane standard was used as the external referencing standard to calibrate the ^13^C chemical shifts.

#### 3.6.5. Folin–Ciocalteu Analysis

The total phenolic content of the lignins was determined by a modified Folin–Ciocalteu (FC) protocol with some modifications to the sample preparation step, as previously described [54,55]. Briefly, the samples were dissolved in DMSO with a final concentration of 2 mg/mL. For each determination, 5 μL of the working solution (or the standard solution) were then mixed with 120 μL of deionized water, 125 μL of FC reagent (Sigma 47641), and kept for 6 min at room temperature after 30 s of vortex stirring. Then, after the addition of 1.25 mL of 5% sodium carbonate and mixing, the vial was incubated in a thermoshaker at 40 °C for 30 min. The reaction mixture absorbance was measured using a UV–Vis spectrophotometer (Jasco V-560) equipped with a temperature-controlled cuvette holder and a thermostatic water bath (Haake K10, Karlsruhe, Germany). All the spectrophotometric measurements were carried out at 760 nm, 25 °C, using a 1 cm optical path cuvette and deionized water as the blank sample. Vanillin was chosen as the reference standard. The calibration curve was constructed with nine different vanillin solutions in DMSO with a concentration in the range 0–500 μg/mL (see Supplementary Materials Figure S2). Each FC assay determination was carried out in triplicate.

#### 3.6.6. ^31^P NMR Analysis

^31^P NMR spectroscopic analyses were recorded on a Bruker Instrument AVANCE400 spectrometer (Milano, Italy). The acquisition and data treatment were performed with Bruker TopSpin 3.2 software (Milano, Italy). The spectra were collected at 29 °C with a 4 s acquisition time, 5 s relaxation delay, and 256 scans. Prior to the analysis, the samples were dried for 24 h under a vacuum and then derivatized according to the following procedure.

The sample (40 mg) was completely dissolved in 300 μL of *N*,*N*-dimethylformamide. To this solution, the following components were added: 200 μL of dry pyridine, 100 μL of solution of an internal standard (10 mg of Endo-*N*-hydroxy-5-norbornene-2,3-dicarboximide (Sigma 226378) dissolved in 0.5 mL of a mixture of pyridine and CDCl_3_ 1.6:1 *v*/*v*), 50 μL of a relaxation agent solution (5.7 mg of chromium (III) acetylacetonate (Sigma 574082) dissolved in 0.5 mL of a mixture of pyridine and CDCl_3_ 1.6:1 *v*/*v*), 100 μL of 2-chloro-4,4,5,5-tetramethyl-1,3,2-dioxaphospholane (Sigma 447536), and, at the end, 200 μL of CDCl_3_. The solution was centrifuged and/or filtered if necessary. All the chemical shifts reported were related to the reaction product of the phosphorylating agent with water, which gave a signal at 132.2 ppm.

#### 3.6.7. Fourier-Transform Infrared Spectroscopy Analysis

The FTIR spectra were collected with a Nicolet Nexus 760 FTIR spectrophotometer. The samples were prepared by pressing the lignin powders with KBr powder to obtain thin discs. The spectra were recorded at room temperature, in air, in transmission mode (64 accumulated scans at a resolution of 4 cm^−1^) in the 4000–1000 cm^−1^ wavenumber range.

#### 3.6.8. Differential Scanning Calorimetry Analysis

DSC analysis was employed to investigate the thermal transitions in the lignin samples. Measurements were carried out on 10–15 mg samples by means of a Mettler-Toledo DSC 823e instrument. Three runs (heating/cooling/heating) were performed: from 25 °C to 150 °C to remove the water from the samples, from 150 °C to 25 °C, and from 25 °C to 200 °C, at a scan rate of 20 °C/min under a nitrogen flux. The glass transition temperature (Tg) of the samples was evaluated as the inflection point in the second heating run.

#### 3.6.9. Thermogravimetric Analysis

TGA was performed on all the lignin samples (~15 mg) by means of a Q500 TGA system (TA Instruments, Milan, Italy) from an ambient temperature to 800 °C, at a scan rate of 10 °C/min in air.

### 3.7. Determination of Water Reduction in Cement Pastes

The complete description of the laboratory method for the preliminary estimation of the water reduction capabilities of the fractionated lignins was reported in detail in a previous work [15]. Briefly, the analysis is based on the evaluation of the relative Bingham dynamic yield torque of cement pastes containing 0.2% of lignin (*w*/*w* relative to dry cement) at a different water/cement (*w*/*c*) ratio [56].

By measuring the dynamic yield torque of the cement pastes at various *w*/*c* ratios, it is possible to calculate by inverse regression the *w*/*c* ratio needed to obtain a predetermined reference torque value and thus to determine the water reduction capacity for each lignin under study. In this study, the reference Bingham dynamic yield torque was obtained with a pure cement paste with a 0.45 *w*/*c* ratio. The actual water reduction obtained for each lignin is given by the following equation

$$W_{R}=100\;\cdot \;(1-\frac{W_{L}}{W_{ref}})$$

where *W_R_* is the water reduction (%), *W_L_* is the measured *w*/*c* ratio at the reference yield torque for the given lignin, and *W_ref_* is the reference *w*/*c* ratio (0.45). This procedure allows us to measure the effective water reduction capability of each lignin in the preparation of cement pastes in the given conditions. In the present work, the water reduction capability was measured for rRH-Lignin, for pRH-Lignin and, as a reference, for a well-known commercial soda lignin (Protobind).

## 4. Conclusions

Among the agri-food cultivations, rice has a huge social and economic importance. For this reason, the exploitation of waste residues coming from its production possesses an important interest for the scientific community and for the society.

In this work, we demonstrated a multistep lignocellulosic fractionation process of raw and parboiled rice husks by means of DESs based on non-toxic, renewable components. The fractionation allowed us to recover a cellulose/silica fraction, precipitated by the addition of ethanol, and a lignin-based fraction, precipitated by the addition of water.

The lignin fractions obtained from both raw and parboiled rice husks were fully characterized and compared for the first time. The results show similar physico-chemical properties between the two recovered lignins, indicating that the parboiling process does not alter significantly the lignin characteristics. Moreover, because of the mild separation conditions allowed by the DES-mediated process, the resulting lignins were underivatized and demonstrated a limited degradation.

Both lignins were studied as a potential base for the production of cement water reducers, the latter being an important application field for lignin derivatives. The obtained results confirmed the substantial equivalence of the two lignins for the target application and indicated that both are comparable to a commercial soda lignin taken as a reference benchmark.

A preprocess step based on fermentation was also studied. However, the obtained results demonstrated the limited degradability of both raw and parboiled RHs, even using cellulose-degrading fungi. A possible cause for such behavior was found in the relatively high silica content in both RHs (about 13%).

These preliminary data show, at a 25 g lab-scale, the potentialities of DES-mediated fractionation processes in the lignocellulosic fractionation of RHs. This protocol is a promising base for a future implementation on a larger scale. Considering the very large volume of the worldwide rice production and the environmental friendliness of DES-based processes, the reported results constitute an encouraging step towards the exploitation of RH wastes in line with the strategic objectives of the circular economy.

## Figures and Tables

**Table 1:** Composition of the studied RHs biomasses.

Sample	Hemicellulose (% *w*/*w*)	Cellulose (% *w*/*w*)	Silica (% *w*/*w*)	Lignin (% *w*/*w*)	Soluble Fraction (% *w*/*w*)
rRH	19.5	43.2	12.9	19.2	5.20
pRH	27.3	36.7	12.4	18.5	5.10

**Table 2:** Relative abundance of the monosaccharides deriving from RHs hemicellulose hydrolysis.

RH	Rhamnose	Fucose	Arabinose	Xylose	Mannose	Glucose	Galactose
rRH	0.4	0.5	49.0	44.9	-	1.2	4.0
pRH	0.2	0.5	22.6	69.2	-	4.9	2.6

**Table 3:** List, composition, and density (measured at 18 °C) of prepared DESs.

DES Number	Composition DES HBA/HBD	Molar Ratio (HBA/HBD)	Density of Pure DES (g/cm^3^)
1	Choline chloride/Acetic acid	1/2	1.10
2	Choline chloride/L-Lactic acid	1/5	1.18
3	Betaine Glycine/Acetic acid	1/2	1.11
4	Betaine Glycine/L-Lactic acid	1/5	1.20

**Table 4:** Number average molecular weight (M_n_), weight average molecular weight (M_w_), and polydispersity index (*Ð*) of all examined lignins (samples were eluted after acetylation; reported values are relative to polystyrene standards).

Sample	M_n_ (g/mol)	M_w_ (g/mol)	*Ð*
rRH-Lignin	1380	5330	3.86
rRH_T_-Lignin	1360	5195	3.82
pRH-Lignin	1320	3930	2.98
pRH_T_-Lignin	1310	3860	2.95
Protobind 1000	830	2800	3.37

**Table 5:** Results of the total reducing sugar quantification.

Sample	Reducing Sugars/Biomass (*w*/*w*%)	Reducing Sugars/Biomass after Hydrolysis (*w*/*w*%)
rRH-Lignin	0.19	5.7
rRH_T_-Lignin	0.51	9.2
pRH-Lignin	0.31	9.1
pRH_T_-Lignin	0.38	14
Protobind 1000	0.34	13

**Table 6:** Results of the determination of phenolic hydroxyl groups expressed as vanillin equivalents/g of lignin sample. Estimated standard errors ± 0.1 mmol/g vanillin equivalent (1σ, from calibration data).

Sample	Vanillin Equivalent Content (mmol/g)
rRH-Lignin	1.6
rRH_T_-Lignin	1.3
pRH-Lignin	1.6
pRH_T_-Lignin	1.7
Protobind 1000	3.1

**Table 7:** Detailed hydroxyl/carboxyl quantification by ^31^P NMR (as mmol of functional group per g of dry lignin).

Sample	–OH Aliphatic (mmol/g)	–OH Aromatic (mmol/g)	–COOH (mmol/g)
rRH-Lignin	2.42	7.12	1.43
rRH_T_-Lignin	2.48	7.10	1.02
pRH-Lignin	3.01	7.77	1.42
pRH_T_-Lignin	3.08	7.49	1.36
Protobind 1000	3.00	4.67	1.42

**Table 8:** Characteristic mass loss temperatures (10% mass loss, 50% mass loss, and maximum degradation rate) for the analyzed lignins. The reference Protobind system is also reported.

Sample	T_10%_ (°C)	T_50%_ (°C)	T_DTGmax_ (°C)
rRH-Lignin	247	480	532
rRH_T_-Lignin	256	482	531
pRH-Lignin	245	477	538
pRH_T_-Lignin	243	486	538
Protobind 1000	227	444	472

## Data Availability

Data are contained within the article and the Supplementary Materials.

## Supplementary Materials

The following supporting information can be downloaded at: https://www.mdpi.com/article/10.3390/molecules27248879/s1, Figure S1: ^1^H NMR spectrum of DES 2 choline chloride/L-lactic acid (1:5 mol/mol); Figure S2: Calibration curve of Folin–Ciocalteu phenol titration with vanillin.; Table S1: Yields of mass recovery in the different tested DESs.
