# Supplementary material for: Fractionation of Raw and Parboiled Rice Husks with Deep Eutectic Solvents and Characterization of the Extracted Lignins towards a Circular Economy Perspective
Source: Molecules. 2022 Dec 14;27(24):8879. doi: 10.3390/molecules27248879 (PMC9785053; doi:10.3390/molecules27248879)
Supplement: Supplementary file 1 [file molecules-27-08879-s001.zip › molecules-2010475-supplementary.pdf]

Supplementary Materials

Figure S1. ^{1}H NMR spectrum of DES 2 choline chloride/L-lactic acid (1:5 mol/mol). 2

Figure S2. Calibration curve of Folin-Ciocalteu phenol titration with vanillin. 3

Table S1: Yields of mass recovery in the different tested DESs. 4

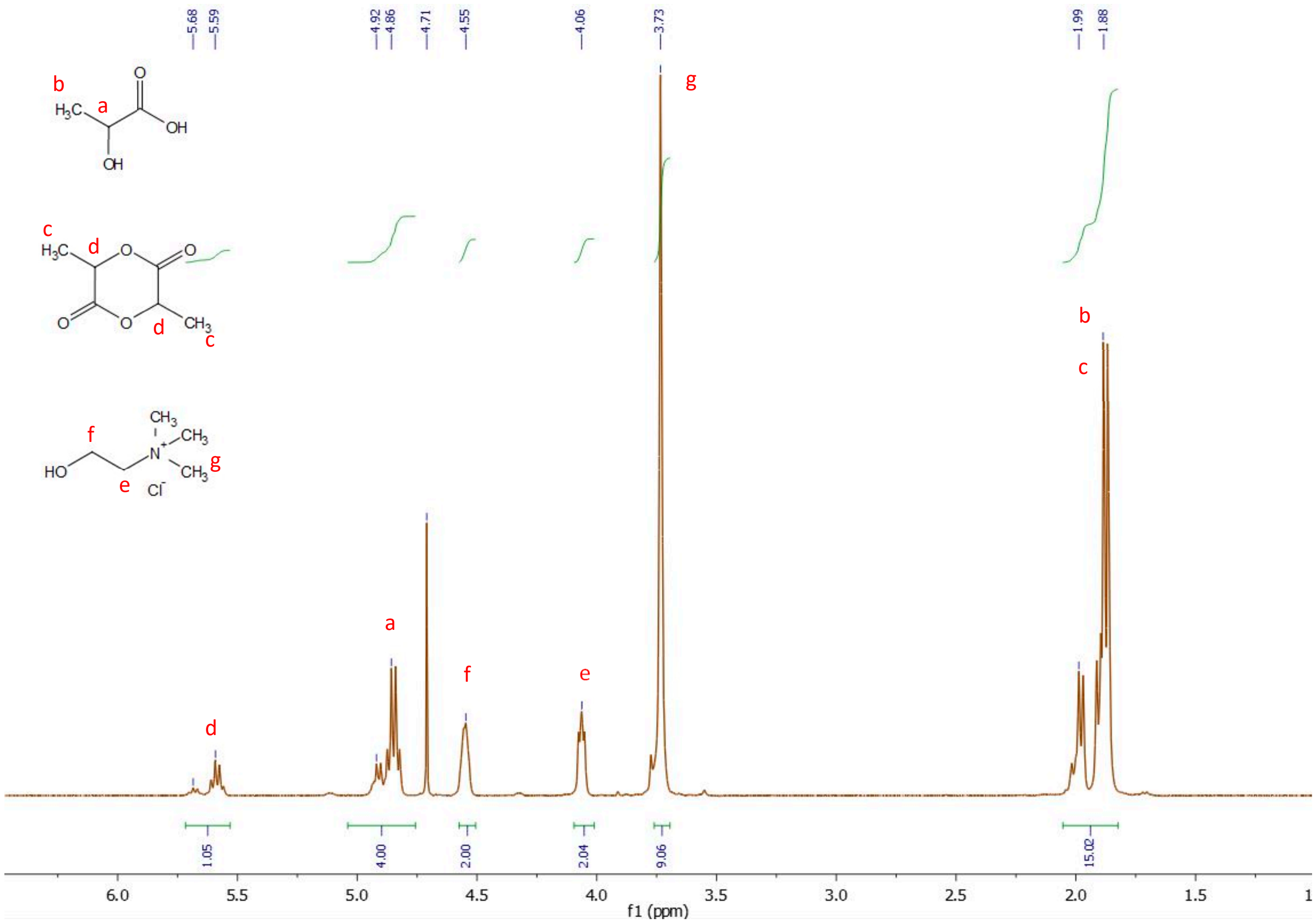

Figure S1. ^{1}H NMR spectrum of DES 2 choline chloride/L-lactic acid (1:5 mol/mol).

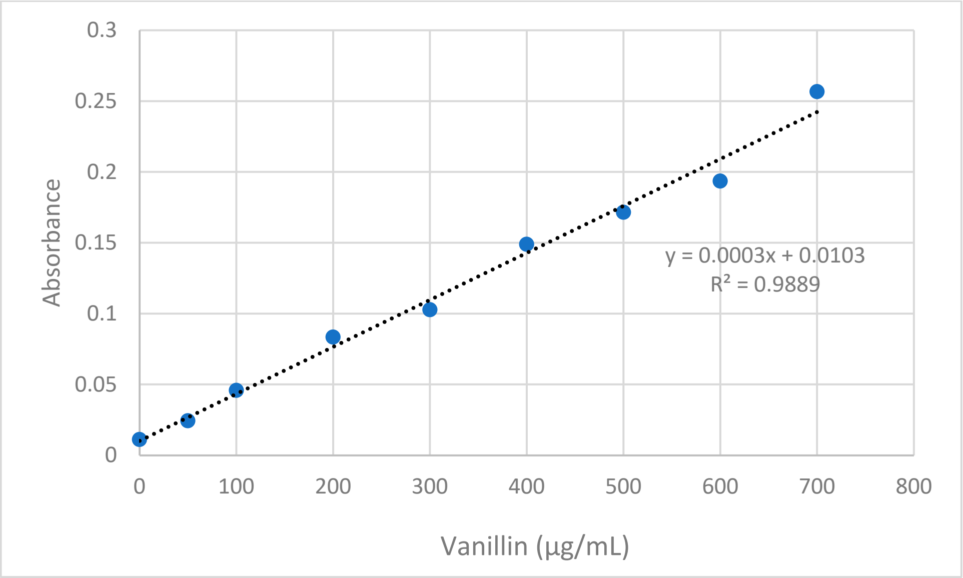

Figure S2. Calibration curve of Folin-Ciocalteu phenol titration with vanillin.

DES	Cellulose-enriched fraction from wRH (% w/w Biomass)	Lignin fraction from wRH (% w/w Biomass)	Cellulose-enriched fraction from pRH (% w/w Biomass)	Lignin fraction from pRH (% w/w Biomass)
1	-	-	65.2 (with silica)	8.02
2	46.6	15.9	47.2 (without silica)	14.5
3	-	-	-	-
4	-	-	70.1 (with silica)	3.64

Table S1. Yields of mass recovery in the different tested DESs.

(NB: "-" indicates no results in terms of biomass fractionation).
